# Internal stress induced natural self-chemisorption of ZnO nanostructured films

**DOI:** 10.1038/srep43281

**Published:** 2017-02-24

**Authors:** Po-Wei Chi, Chih-Wei Su, Da-Hua Wei

**Affiliations:** 1 Institute of Manufacturing Technology and Department of Mechanical Engineering, National Taipei University of Technology (TAIPEI TECH), Taipei 10608, Taiwan

## Abstract

The energetic particles bombardment can produce large internal stress in the zinc oxide (ZnO) thin film, and it can be used to intentionally modify the surface characteristics of ZnO films. In this article, we observed that the internal stress increased from −1.62 GPa to −0.33 GPa, and the naturally wettability of the textured ZnO nanostructured films changed from hydrophobicity to hydrophilicity. According to analysis of surface chemical states, the naturally controllable wetting behavior can be attributed to hydrocarbon adsorbates on the nanostructured film surface, which is caused by tunable internal stress. On the other hand, the interfacial water molecules near the surface of ZnO nanostructured films have been identified as hydrophobic hydrogen structure by Fourier transform infrared/attenuated total reflection. Moreover, a remarkable near-band-edge emission peak shifting also can be observed in PL spectra due to the transition of internal stress state. Furthermore, our present ZnO nanostructured films also exhibited excellent transparency over 80% with a wise surface wetting switched from hydrophobic to hydrophilic states after exposing in ultraviolet (UV) surroundings. Our work demonstrated that the internal stress of the thin film not only induced natural wettability transition of ZnO nanostructured films, but also in turn affected the surface properties such as surface chemisorption.

Recently, the surface wettability of solid materials has been a promoted and demonstrated character for many promising applications such as environmental cleanup, optoelectronic devices, and gas sensors^1^,^2^,^3^,^4^,^5^. Therefore, many research works focused on the surface wetting behavior of solid materials is particularly required and important. In addition, it is well-known that the chemical identity of a surface controls its wetting behavior such as carboxylic and hydroxyl functional groups constructing hydrophilic surfaces, while alkyl and perfluoroalkyl groups render surfaces in hydrophobicity^6^. Moreover, as demonstrated by Wenzel, Cassie and Baxter in the 1930s and 1940s^7^,^8^, surface morphology including micro- and nanostructures was found to play a key role in determining its wetting behavior. In general, the materials with hydrophobic surface are usually fabricated by the modifying of their surface for forming nanoscaled structures with low surface energy. As an oxide semiconductor, ZnO thus becomes an excellent candidate because (002) plane is the lowest surface energy plane and easier to form nanostructure. Up to now, many research works have been reported to enhance the wetting behavior of the ZnO with the superhydrophobic surface by forming different types of nanostructures. Li *et al*. prepared hydrophobic ZnO films with cotton-like nanostructures by hydrothermal method with various buffer layers^9^. Kenanakis *et al*. synthesized ZnO wurtzite microrods and flower-like nanostructures on glass and ITO substrates by the aqueous chemical growth (ACG) technique, and the surface wettability exhibited in hydrophobic state^10^. Over the past research works, most of research groups have focused on one-dimensional (1-D) materials formed by chemical synthesis method, and there is still quite limited research work about the two-dimensional (2-D) nanostructured ZnO films directly obtained by physical method. The magnetron sputtering technique has attracted great interest because of its advantages for film growth with controllable characteristics, such as the orientation in the crystalline is closed to epitaxial growth at relatively low temperature. These properties are mainly caused by the kinetic energy of the particle bombardment from the plasma during the thin film deposition processes. The plasma energy enhanced the surface migration effect and surface bonding state. These energetic particles bombardment can produce large internal stress in the thin film, and it can be used to intentionally modify thin film surface properties^11^,^12^. Although Lin *et al*. reported that surface-modified ZnO films sputtered onto glass substrates exhibited in hydrophobic state with contact angle of 101°, but the natural surface wettability enhancement of intrinsic ZnO films has been still seldom studied^13^. However, the relationship between the wettability and internal stress of ZnO films is still not clear enough. On the other hand, ZnO is a general compound for common photocatalysts among the metal oxides, such as it is suitable to react under UV light due to its large band gap^14^, and its wettability can be modulated obviously by irradiation with UV light^15^,^16^. Above results indicate that the functional ZnO material is a good choice for self-cleaning coating. Until now, many research works have been reported about the hydrophobic/hydrophilic reversible process caused by light-controlled method for ZnO-based nanostructures^17^,^18^,^19^,^20^. ZnO has direct wide band gap (3.37 eV), high exciton binding energy (60 meV) and optical transparency in the visible light, thus, ZnO is an expected material for many novel applications, for example, piezoelectric transducers, transparent thin film transistors, chemical biosensors^21^,^22^, solar cells and ultraviolet (UV) detectors^23^,^24^,^25^. According to the above functionalities, ZnO can provide a hydrophobic surface, which may be transformed to hydrophilic surface by UV irradiation, coexists with intrinsic semiconductor properties and a particular surface morphology. As for the surface chemical property of ZnO compound, the tunable and reversible wettability can be explained by the competition results between desorption of organic chains and hydroxyl groups rearrangement on the material surface. However, the surface wetting properties of ZnO nanostructures are highly developed and expected to exhibit more advanced controllable wettability including a rapidly hydrophilic/hydrophobic switched behavior with switchable contact angles.

In this present article, the textured wurtzite ZnO nanostructured films with tunable wettability associated with variable internal stress state were directly deposited onto glass substrates at room temperature by RF sputtering system. The natural wetting behavior can be attributed to the controllable internal stress state of ZnO that caused the different content of self-chemical adsorption of hydrocarbons onto the ZnO surface. In addition, the relationship between the surface compositions and the orientation of interfacial water molecules were also confirmed. Furthermore, the transparency for ZnO nanostructured films was also examined. The surface wettability for all ZnO thin films was measured by water contact angle measurement. The switchable wettability was explored by varied the conditions of UV exposure and dark room surroundings storage. Our work also claimed a straightforward way to prepare textured ZnO films with tunable surface chemistry by controlling a factor of internal stress state. It not only extends the potential applications of ZnO textured thin films but also assists a profound understanding with material design and devices development on commercial glass substrates.

## Results and Discussion

There are many developing methods in order to realize the internal strain/stress of the thin film. Zheng *et al*. measured internal strain/stress of flat TiO_2_ films by using the substrate bending method, indicating presence of a high compression stress in TiO_2_ films due to the high deposition rate and low deposition temperature in sputtering process^26^. Sahoo *et al*. studied the internal stress of ZnO nanowires by Raman spectrum, and they confirmed the bent ZnO nanowire formed under tensile strain^27^. Hisao *et al*. used the XRD analysis to calculate internal strain/stress of *L*1_0_ FePt films, and claimed the effect of stress on phase transition of *L*1_0_ FePt metal alloy^28^. Among all methods, XRD analysis is the most convenient way to determine the impact of the internal strain/stress in 1-D nanowires and 2-D thin films.

**Figure 1:**
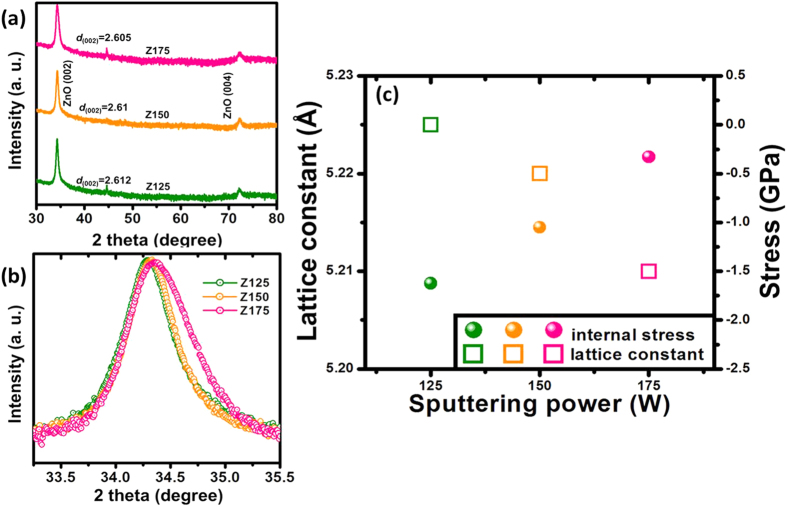
(**a**) XRD patterns for the samples Z125, Z150 and Z175, respectively. (**b**) The corresponding slow scan curves of the ZnO (002) peak in the *θ*-2*θ* scan ranged from 2*θ* = 33 to 35.5°. (**c**) Lattice constants and the internal stress of the samples as a function of sputtering power.

Figure 1(a) shows the X-ray diffraction patterns for the ZnO nanostructured films sputtered onto glass substrates with RF powers of 125, 150 and 175 W, respectively. XRD patterns show the samples exhibit a strong peak located at around 2*θ* = 34°, which is corresponded to the ZnO (002) plane (JCPDS Card: 36-1451). In addition, no peaks except the (00*n*) diffraction peaks are observed in the whole diffraction patterns (*θ*-2*θ* scan), indicating the highly textured ZnO films with preferred *c*-axis orientation and belong to a hexagonal wurtzite structure. The strong (002) diffraction peak intensity from the ZnO (002) plane is due to its lowest surface energy in the (002) basal plane from ZnO phase, leading to a preferred orientation along the [001] crystalline direction. On the other hand, from the slow scan curve as shown in Fig. 1(b), it can be observed that the (002) diffraction peak of samples clearly shifted from low angle side (2*θ* = 34.29°) to high angle side (2*θ* = 34.39°) as the change of plasma energy from 125 W to 175 W. Reality as presented by Ghosh *et al*.^29^, the strain normal to the substrate is given by (*c*_*0*_ − *c*)/*c*_*0*_, where *c* is the lattice constant obtained from strong (002) diffraction peak in the XRD patterns, *c*_*0*_ is the corresponding value for bulk ZnO, is 5.206 Å^30^. However, for materials in hexagonal crystal structure, the linear stress components can be described as:


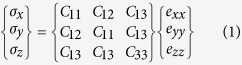


where *C*_*ij*_ are the elastic stiffness constants and *e*_*ij*_ is the linear strain in the *i*th direction.

As purposed by Maniv *et al*.^31^, the stress σ_z_ is zero in the films except very close to the edge. Therefore,


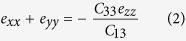


The stress in the plane can be displayed as:


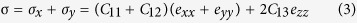


combined the Eqs 2 and 3, the Eq. 4 can be written as:


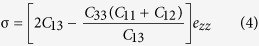


and *e*_*zz*_ is the strain normal to the substrate, which is obtained from measured lattice constant. Thus,


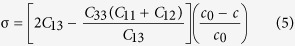


Substituting the values of the stiffness constants for ZnO in Eq. 5 with are *C*_11_ = *C*_33_ = 210 GPa, *C*_12_ = 120 GPa and *C*_13_ = 105 GPa^32^,^33^, the internal stress σ can be written by the following formula:


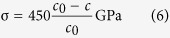


As presented in Eq. 6, it is necessary to calculate the lattice constant for each ZnO nanostructured film. From Fig. 1(a), the diffraction peaks for three samples were located at 2*θ* = 34.29°, 34.33° and 34.39°, following the Bragg’s formula, the lattice constant can be calculated.


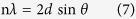


where λ is X-ray wavelength and *d* is interplanar spacing of ZnO (002) crystal plane. After substituting *θ* which is measured by XRD for each sample, the values of *d*_(002)_ for ZnO nanostructured films are 2.612, 2.610 and 2.605, respectively. After all typical definition, substituting the value of *d*_(002)_ of each sample to the following formula^34^:


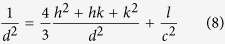


where *h* = 0, *k* = 0, *l* = 2 for ZnO (002) diffraction peak, therefore, the values of lattice constant *c* for three samples are 5.225 Å, 5.221 Å and 5.211 Å. Finally, substituting the lattice constant *c* of each sample to Eq. (6), then the internal stress of the ZnO nanostructured films can be calculated. The internal stress of the ZnO nanostructured films was calculated by using XRD method as shown in Fig. 1(c). Obviously, the stress values of sample Z125, Z150 and Z175 are −1.62 GPa, −1.05 GPa and −0.33 GPa as the tunable of the applied sputtering power ranged from 125, 150 to 175 W, respectively.

**Figure 2:**
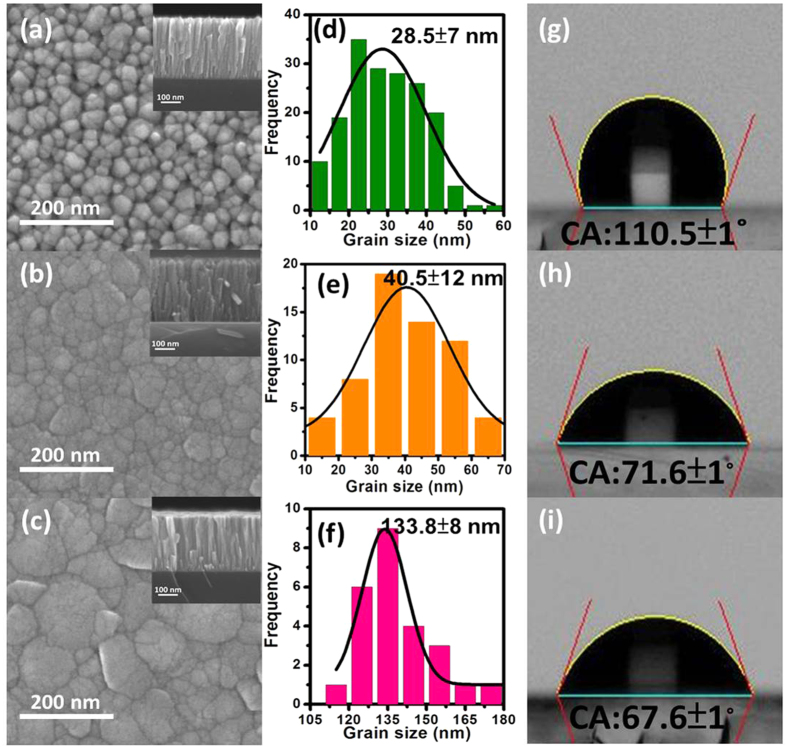
Top view FE-SEM images for the ZnO nanostructured films (**a**) Z125, (**b**) Z150 and (**c**) Z175, respectively. (**d**–**f**) show the grain size histograms for evaluating average size and its distribution of the samples Z125, Z150 and Z175, respectively. (**g**–**i**) are the corresponding CA images for the samples Z125, Z150 and Z175, respectively.

As the above mentioned, the transformed internal stress is found while ZnO nanostructured films deposited at higher sputtering power. This transition is attributed to ion bombardment during the sputtering process. As reported by Ditchfield *et al*.^35^,^36^ and Tolstova *et al*.^37^, the higher ion bombardment of plasma energy can lead to relaxation of internal stress and grain boundaries efficiently, indicating an obvious variation in total Gibbs free energy. At the same time, the different microstructure induced by internal stress can be observed as depicted in the inset of Fig. 2(a–c), moreover, ion bombardment also assists the phase transition of metal-based materials^38^.

Figure 2(a–c) are the top view FE-SEM images for the ZnO nanostructured films with varied internal stress of −1.62 GPa, −1.05 GPa and −0.33 GPa, respectively. The different grain structure was induced by varying stress state during the sputtering process that can be easily manipulated. Moreover, a clearly column structure also can be observed from cross-section FE-SEM images as shown in the inset of Fig. 2(a–c). The nanograin structure of ZnO thin films can be observed at compressive stress state (−1.62 GPa) as shown in Fig. 2(a). On the other hand, the morphology of sub-micrograin can be observed while the internal stress increased from −1.62 GPa to −1.05 GPa as shown in Fig. 2(b). Furthermore, the micrograin structure of ZnO thin films can be observed at nearly stress-free state (−0.33 GPa) as shown in Fig. 2(c). This grain type transition can be attributed to the ion bombardment of high plasma energy, which directly leaded to the grain growth. Figure 2(d–f) are the corresponding grain size histograms for evaluating average size and its distribution of the ZnO nanostructured films sputtered onto glass substrates at room temperature with varied internal stress of −1.62 GPa, −1.05 GPa and −0.33 GPa. The grains size in fraction of sample Z125 with internal stress value of −1.62 GPa ranged from 21.5 to 35.5 nm, with an average size of 28.5 ± 7 nm as shown in the Fig. 2(d), which was denoted as nanograin. The grains size in fraction of sample Z150 with internal stress value of −1.05 GPa ranged from 28.5 to 52.5 nm, with an average size of 40.5 ± 12 nm as shown in the Fig. 2(e), which was denoted as sub-micrograin. The grains size in fraction of sample Z175 with internal stress value of −0.33 GPa ranged from 125.8 to 141.8 nm, with an average size of 133.8 ± 8 nm as shown in the Fig. 2(f), which was denoted as micrograin. Above results indicate that the microstructure of the ZnO thin films could be controlled through manipulating the internal stress. The corresponding photos of surface water contact angle are shown in Fig. 2(g–i), respectively. The values of surface water contact angle (CAs) are 110.5 ± 1°, 71.6 ± 1° and 67.6 ± 1° for each ZnO thin film with varied internal stress state from the compressive to the nearly stress-free. The hydrophobic wetting behavior for ZnO film occurred at compressive stress state, which was due to internal stress induced nanograin structure, meanwhile, there were many apertures on nanograin surface that provided trapping of air to form air-pocket, more air-pocket can effectively reduce the contact area between water and smooth surface as shown in Fig. 2(g). While the internal stress increased from −1.62 GPa to −1.05 GPa, the smaller contact angle value was measured at around 71.6 ± 1° as shown in Fig. 2(h), moreover, while the internal stress state of ZnO nanostructured films is nearly stress-free, the lowest contact angle value was measured at around 67.6 ± 1° as shown in Fig. 2(i), it was due to internal stress relaxation induced the grain growth from nanograin to micrograin. This transformed process caused the CA decreased, meanwhile, there are many bigger apertures on sub-micrograin surface and liquid may completely penetrate into surface hollows, leading air-pocket loss^39^,^40^, therefore, the hydrophilic wetting behavior for ZnO nanostructured films occurred at nearly stress-free state (−0.33 GPa). For the purpose of understanding the microstructural features varied boundary from nanograin to micrograin, the obviously different internal stress state for samples Z125 and Z175 effectively induced various surface wetting behavior, the surface morphology, chemical adsorption and optical property of ZnO nanostructured films will be mainly discussed below.

**Figure 3:**
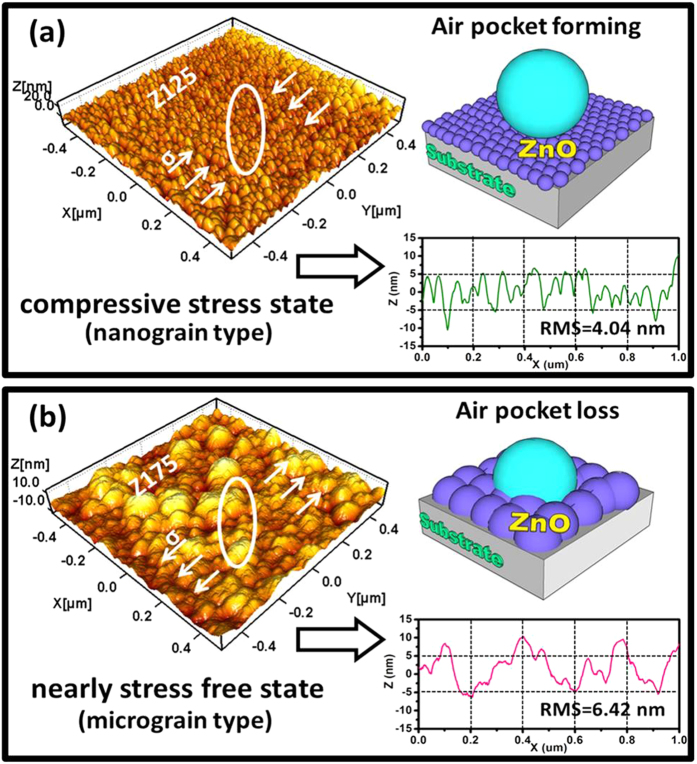
(**a**,**b**) show atomic force microscope (AFM) 3-D images with scanning size of all domain images fixed at 1 × 1 μm^2^, and the corresponding schematic diagrams and line profiles illustrating the ZnO nanostructured films with different types of grain structure for the samples Z125 and Z175, respectively.

The corresponding three-dimensional (3-D) AFM images (1 μm × 1 μm) are shown in Fig. 3(a,b), respectively. The 3-D surface topography images show the tendency of the surface roughness for ZnO thin films increased as the internal stress increased. The average surface roughness values (root-mean-square, RMS) of the ZnO nanostructured films are 4.04 nm and 6.42 nm for Z125 and Z175 samples, respectively. According to the above results, it could be understood that the wurtzite ZnO nanostructured films could provide the nanoscaled surface roughness to support the air-pocket formation, which can efficiently store the air. Such kinds of the ZnO nanostructured films could be easy to form by tuning the different internal stress state of the film, and it provided a convenience way to form nanometer-scaled structure for multifunctional ZnO based electronic nanodevices^41^,^42^. The schematic diagrams as shown in Fig. 3(a,b) are illustrating for the different models for the state of water droplet onto various internal stress state of the ZnO thin films. In addition, the aforementioned apertures on the surface of ZnO nanostructured film can be further investigated through the line profile of the AFM cantilever as shown in the insets of Fig. 3(a,b). From the line profile of the AFM cantilever of each ZnO nanostructured film, the average size of apertures and its distribution size can be easily evaluated (see in the Supplementary Information). As shown in Figure S2(a), the aperture size in fraction of sample Z125 with internal stress value of −1.62 GPa ranged from 37.3 to 48.1 nm, with an average size of 42.7 ± 5.4 nm, indicating that when the ZnO nanostructured films presented in compressive stress state (Z125), the nanograin stacked together and formed apertures that could provide the trapping air and become air-pocket, causing the composite solid–air–liquid interface and lead surface wettability in hydrophobicity as shown in Fig. 3(a). On the other hand, as shown in Figure S2(b), the aperture size in fraction of sample Z175 with internal stress value of −0.33 GPa ranged from 64.5 to 108.9 nm, with an average size of 86.7 ± 22.2 nm, meaning that while the internal stress relaxation (Z175) occurs, the bigger apertures exist on film surface and liquid may easily penetrate into hollow site of the surface, leading air-pocket loss, therefore, the hydrophilic wetting behavior for ZnO nanostructured films occurred as shown in Fig. 3(b). Above results indicated that the process of air-pocket forming and losing can be easily observed by 3-D AFM images, and it is the direct evidence to explain natural surface wetting behavior of ZnO nanostructured films.

**Figure 4:**
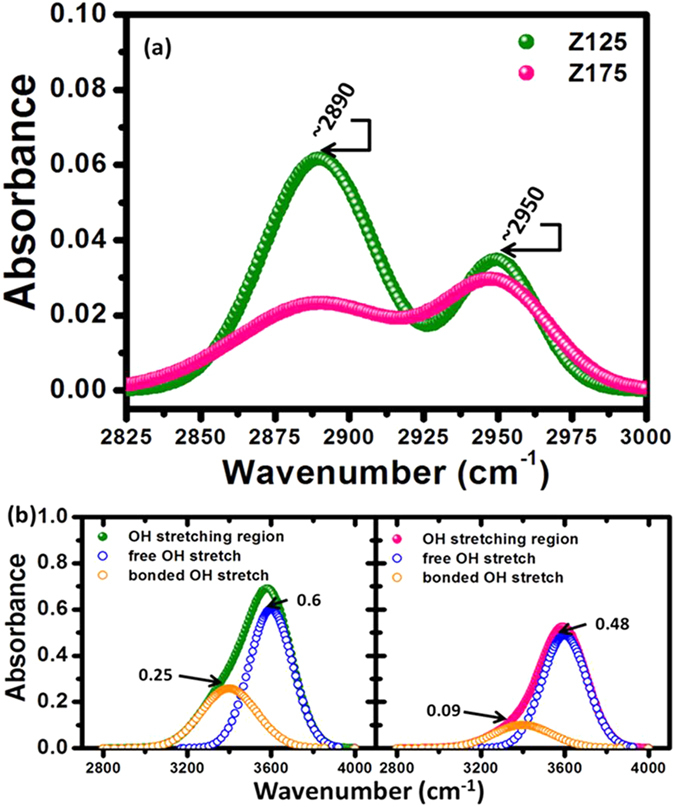
FTIR/ATR absorbance spectra recorded in the (**a**) 3000–2825 cm^−1^ and (**b**) 4050–2750 cm^−1^ spectral wavelength range for the samples Z125 and Z175, respectively.

In addition, there is another reason for the hydrophobic state of the ZnO nanostructured films that can be related to the adsorption of organic molecules. Chemical and physical adsorption behavior on the surface of various high energy materials is often driven by the tendency to reduce the total Gibbs free energy of the system by diminishing the surface energy part^43^,^44^,^45^,^46^. In order to realize the adsorption of organic contaminants on the surface from the environment, Fourier transformed infrared (FTIR) absorption spectra were measured. As shown in Fig. 4(a), the characteristic alkyl C-H bond stretching vibrations of CH_2_ and CH_3_ groups were detected from samples in the range between 3000 and 2800 cm^−1^. According to the absorption intensity of the spectra, indicating that the surface of ZnO nanostructured film in compressive stress state can adsorb more hydrocarbon contaminants from air, and this is another reason to explain why the hydrophobic surface formed. On the other hand, as suggested by Scatena *et al*.^47^ and Azimi *et al*.^48^, the hydrophobic surface usually accompanied a strong OH bonded associated with the tetrahedral structure of bulk water molecules, thereby the Fourier transform infrared/attenuated total reflection (FTIR/ATR) spectroscopy was applied to confirm the orientation of interfacial water molecules as shown in Fig. 4(b). The broad band of vibrational spectrum of water between 3000 and 3800 cm^−1^ (the OH stretching region), which was yielded against the background spectrum of the clean diamond with the air, and then deconvoluted into peaks located at around 3400 and 3600 cm^−1^. The peaks located at 3400 and 3600 cm^−1^ are used to present the OH stretching modes in hydrogen-bonded OHs (bonded OH stretch) crossed over the interface and non-hydrogen-bonded OHs (free OH stretch) pointed on the surface, respectively. In general, the peak at 3600 cm^−1^ has a higher intensity at the surface of hydrophobic materials than that of hydrophilic ones. From the FTIR/ATR spectroscopy, it can be observed that the intensity of the peak at 3600 cm^−1^ for Z125 is 1.2 times higher than that of Z175. Moreover, the peak at 3400 cm^−1^ also has a higher intensity for sample Z125 compared with sample Z175, indicating that the former surfaces have a higher number of OHs crossed over the interface while the internal stress relaxation is favored in ZnO thin film.

**Figure 5:**
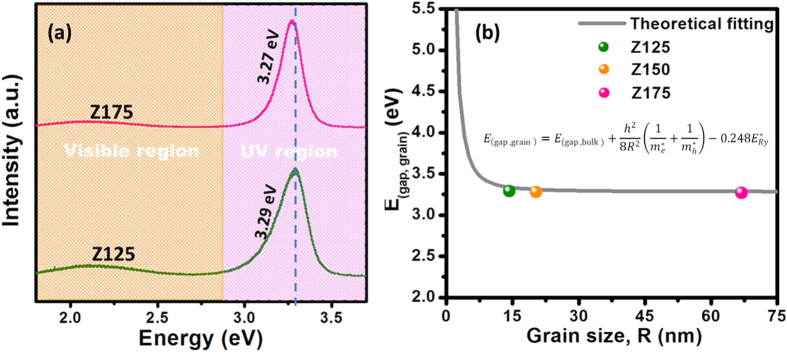
(**a**) Photoluminescence spectra for the samples Z125 and Z175 W, respectively. (**b**) Plot of *E*(gap, grain) obtained from PL measurement as a function of the grain size.

To confirm further the optical absorption of the ZnO films by photoluminescence (PL) spectra is thought to be very important for usual approach in the future. Normally, there are two emission bands can be observed from the PL spectra of ZnO nanostructured film at room temperature. The sharp and strong emission in the UV region is in line with the near-band-edge (NBE) emission, and the broad emission in the visible region is attributed to intrinsic defects. Figure 5(a) shows the PL spectra of samples Z125 and Z175, respectively. The samples exhibit a remarkable NBE emission peak located at around 3.3 eV, which is related to the direct recombination of photo-generated charge carriers (excitonic emission). On the other hand, an extremely weak defect band at around 2.2 eV caused by the intrinsic defect such as oxygen vacancies in the ZnO nanostructured films can be observed, indicating ZnO nanostructured films are almost defect-free^49^,^50^. It is interesting that the peak location of NBE emission shifts toward the lower energy side from 3.29 to 3.27 eV as the grain size of ZnO nanostructured films transformed from nano to sub-micro scale. It is worth noting that the energy of the NBE emission peak of ZnO nanostructured film is inferior to that of bulk, which is at 3.37 eV. A similar phenomenon of emission peak shifting in PL and cathodoluminescence (CL) spectra as the size variation effect in ZnO nanostructured films has been confirmed. Chen *et al*. reported the blue-shift of ZnO nanostructures accompanied by changing in size variation at the nanometer scale^49^. Ghosh *et al*. presented that the observed change in the emission energy for ZnO nanostructures as their size increasing and shape variation^51^. So based on previous works^52^,^53^,^54^, the NBE emission peak shifting of the PL spectrum of ZnO nanostructured films in our present work can be attributed to the quantum-size confinement effect. Figure 5(b) shows a plot of grain band gap *E*_(gap, grain)_ versus the grain size. The solid curve is theoretical fit of equation (see in the Supporting Information), while the symbols are the values of grain size estimated from SEM images (Fig. 2(a,c)) and their corresponding band gap energies measured from PL. As shown in Fig. 5(b). It can be observed that the band gap energy for the ZnO nanostructured films with different grain types measured from the PL almost fits the theoretical curve. It is demonstrated that the band gap energy show theoretical variation with the grain size. In addition, our results clearly show a possible band gap tunable effect caused by a key factor of internal stress of film. In fact, the experimental emission peak actually shifts to the low energy or high wavelength as shown in Fig. 5(a). The above discussion leads to the conclusion that the variable grain type induced by different internal stress state of the film and the corresponding NBE emission of the ZnO nanostructured films appears to be related to their grain size on the morphology associated with tunable stress.

**Figure 6:**
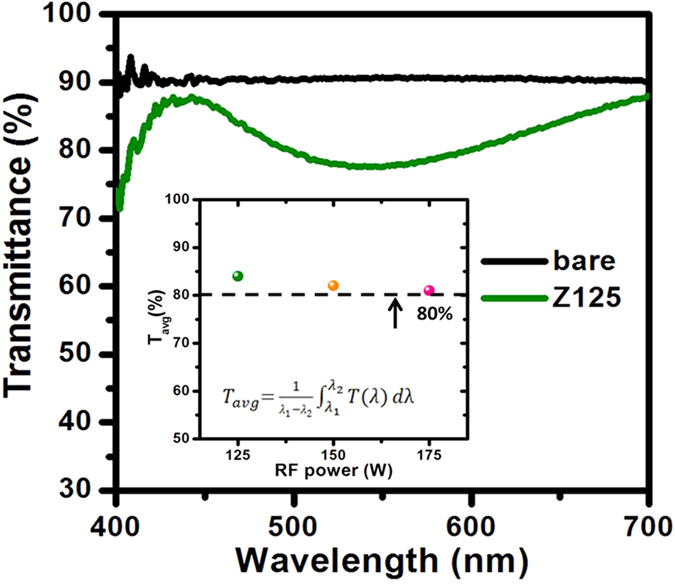
Optical transmittance spectra for the pure-bare glass substrate and the sample Z125. The inset shows the average transmittance for all ZnO nanostructured films.

The high transmittance plays an important role in the particular industrial products such as self-disinfection glass and wearable modern equipment. For understanding the quantitative evaluation of transparency, the average optical transmittance (*T*_*avg*_) is used to estimate the optical property of the thin films deposited onto glass substrates as shown in Fig. 6. The equation to calculate *T*_*avg*_ can be described as:


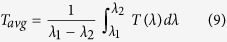


where *T(λ*) is the transmittance of the thin films deposited onto glass substrates, and *λ*_*1*_ and *λ*_*2*_ are the boundary condition of lower limit and upper limit in wavelengths of the optical measurement. As claimed earlier, *T*_*avg*_ was calculated using Eq. (9) over the wavelength range from 400 to 700 nm. The average optical transmittance of the samples Z125, Z150 and Z175 are 84%, 82% and 81%, respectively. On the other hand, the oscillating property of spectrum can be clearly observed and it is due to the formation of uniform ZnO thin films surface and with enough thicker thicknesses. It can be indicated to lead to less light scattering^55^. However, another reason of the oscillator can be attributed to defect electronic states within the band gap associated with oxygen vacancies and interstitial Zn atoms induced inter-band transitions^56^,^57^. In summary, the ZnO nanostructured films with preferred *c*-axis orientation sputtered at room temperature have good transparency and show an average visible light transmittance higher than 80%. It is demonstrated that the hydrophobic (002) textured ZnO nanostructured films have great potential to use in the field such as smart window combined with the optoelectronic devices.

**Figure 7:**
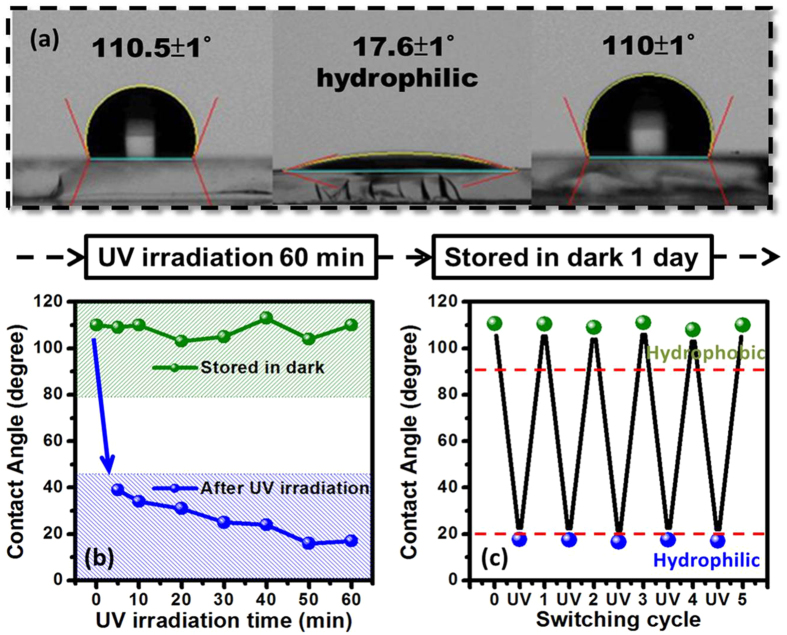
(**a**) Water contact angle (CA) images for the sample Z125 W under UV irradiation with the light wavelength of 365 nm, and stored in dark to reverse to initial state. (**b**) Time dependence of CA for the sample Z125 under ultraviolet (UV) irradiation with the light wavelength of 365 nm. (**c**) Reversible hydrophobicity/hydrophilicity transition of the sample Z125 by alternating UV irradiation and storage in dark surroundings.

As can be seen from the above results and future applications, the sample Z125 shows the best hydrophobicity with CA value of 110.5 ± 1°. In order to control the wettability transition of the ZnO thin films, the sample was put under an ultraviolet (UV) light surroundings with wavelength of 365 nm, which could provide the larger photon energy than the intrinsic band gap (3.37 ev) of the ZnO. The relationship between the ultraviolet (UV) irradiation times varied from 5 to 60 mins and water contact angles (CA) for the sample Z125 as shown in Fig. 7(a–c), respectively. Figure 7(a) showed the corresponding water CA photos accompanied with the measured values for the ZnO (002) nanostructured films, the water droplet spread immediately on the ZnO (002) nanostructured film with nanograin structure after UV irradiation. The sample Z125 was repeatedly recorded after stored in dark surroundings for one day in order to reverse the initial wettability state, it can be observed the CA value of Z125 with UV irradiation for 60 mins clearly changed from 110.5 ± 1° to 17.6 ± 1°. The rapidly decrease of CA value of ZnO film can be attributed to the photocatalystic behavior due to the band gap illumination generates electron-hole pairs in ZnO by photoelectron emission^58^. These electrons and holes can either recombine or move to the surface to react with species adsorbed on the surface. The switchable of wettability transition can also be explained by the following mechanism: via UV irradiation by photon energy, equal or higher than the band gap of ZnO phase, the electrons (*e*^−^) in the valence band are excited to the conduction band. At the same time, the same number of holes (*h*^+^) generated in valence band, some of the holes react with lattice oxygen (O^2−^) or surface oxygen atoms to form surface oxygen vacancies O^1−^, while some of the electrons react with lattice metal ions (Zn^2+^) to form Zn^2+^ defective sites, the water molecules and oxygen may compete with each other to dissociatively absorb on the defective sites. The surface trapped electrons (Zn^+^) tend to react with oxygen molecules adsorbed on the surface. At the same time, the water molecules may act in concert with oxygen vacancy sits (*V*_*O*_), which cause the dissociative adsorption of the water molecules onto the ZnO film surface. The defective sites are kinetically more favorable for hydrophilic hydroxyl groups (OH^−^) adsorption than oxygen adsorption. In general, the oxygen adsorption is favorable in thermodynamic behavior, therefore when the UV irradiated ZnO film was moved to dark surroundings, the oxygen atoms could gradually replace the hydroxyl groups which made the surface come back to its initial state (without UV irradiation) and return its original hydrophobicity. This behavior provides a foundation for photoresponse and the fraction structure enhances CA value. So the UV irradiation can modify the chemical and physical surface states of the ZnO nanostructured film, in turn switching its wettability. On the other hand, further information was carried out from the measurements of time dependence of water contact angles of Z125 as shown in Fig. 7(b). The CA values of ZnO nanostructured film decreased significantly as the UV irradiation time increased, and the surface wettability was switched from hydrophobic to hydrophilic state (blue line). Moreover, after stored in dark surroundings for one day, the ZnO (002) nanostructured films reached the initial/original water contact angle (green line), this suggests that UV irradiation time can be regarded as another key factor of modulation surface wetting behavior. Furthermore, the endurance test of reversible switching behavior between hydrophobicity and hydrophilicity for ZnO nanostructured film is as evidenced and shown in Fig. 7(c). An excellent reversibility of surface wettability can be observed, indicating existence of a stable wetting switching behavior in ZnO nanostructured film. Therefore, the ZnO nanostructured film indeed has a stable recovery state, and is helpful in the applied field such kinds of biosensors and intelligent membranes multifunctional devices, even the *in*-*vivo* or *in*-*vitro* biodevices^59^,^60^.

## Conclusions

The self-assembly ZnO nanostructured films have been successfully sputtered onto glass substrates with preferred *c*-axis orientation by radio-frequency magnetron deposition system at room temperature. The ZnO nanostructured films exhibited good crystallinity and with good visible transparency (higher than 80%). Besides, the ZnO nanograin film had a compressive stress, leading to a morphological evolution. In addition, the compressive stress in ZnO films showed hydrophobic behavior with the highest CA value of 110.5 ± 1°, and the ability of hydrocarbon adsorbates was significantly affected by the internal stress of the film. Moreover, the band gap energy shifting of ZnO nanostructured films with grain size changing has been observed. This kind of band gap energy shifting can be attributed to quantum-size confinement effect. The CA transition of the ZnO nanostructured films could be adjusted from hydrophobic (110.5 ± 1°) to hydrophilic (17.6 ± 1°) induced by the UV light irradiation treatment. The rapid transition of contact angle (CA) value for ZnO nanostructured films can be attributed to the photocatalystic behavior caused by recombination of electrons and holes or move to the surface to react with species adsorbed on the surface via photoelectron emission. The CA transition of the ZnO nanostructured films exhibited an excellent reversibility of surface wettability, indicating a stable wetting switching behavior for ZnO nanostructured film. Therefore, a simple method is presented here that the CA value and switching wettability of 2-D ZnO films can be controlled by the initial stress state via sputtering power and the treatment of UV light irradiation, respectively.

## Methods

### Fabrication of ZnO nanostructured films

ZnO nanostructured films have been sputtered onto the glass substrates by radio-frequency (RF) magnetron deposition system. All the glass substrates have been placed parallel to ZnO ceramic target, which is with 99.99% purity of pressed ZnO powder and the diameter size and thickness are 75 mm and 6 mm, respectively. The distance between target and substrate is 150 mm. Before loaded the substrates into the chamber, the substrates were rinsed in deionized water, ultrasonically cleaned in ethanol and acetone to remove organic contamination and then dried in hot air. The vacuum chamber was pumped down to a base pressure of 4 × 10^−7^ torr, and the argon was filled into chamber sequentially with the low working pressure of 5 × 10^−3^ torr. The internal stress/strain state was manipulated by varying RF powers of 125, 150 and 175 W, and these thin films were named as Z125, Z150 and Z175 respectively. The deposition rates of each RF power were 5.6, 6.5 and 7.2 nm/min, respectively. All samples are with a total nominal thickness of 300 nm onto glass substrates without any buffer layer.

### Characterizations

Fourier transformed infrared (FTIR) spectroscopy with a grazing angle of 80°, 800 scans and 4 cm^−1^ resolution was used to analyze the organic contamination of the film surface, and the interfacial structure of water molecules near the surface of ZnO nanostructured films was also identified by FTIR in attenuated total reflection (ATR) mode with an incidence angle of 45°. The crystalline structure of ZnO films was characterized by *ex*-*situ* X-ray diffraction (XRD) with Cu Kα radiation (λ = 1.54 Å) ranged from 30° to 80° (2*θ*). The surface morphology for the ZnO films was observed by field emission scanning electron microscopy (FE-SEM). The surface topography and corresponding roughness values for ZnO films were further analyzed from the atomic force microscope (AFM) with scan range of 1 μm × 1 μm under tapping mode at room temperature. The photoluminescence spectra (PL) of all samples were obtained using a 325 nm continuous-wave He-Cd laser as the excitation source with a 2400 grooves/mm grating in the backscattering geometry. All PL measurements were carried out at room temperature. UV-Vis-NIR spectrophotometer was recorded at room temperature to study the optical properties of ZnO thin films. The wettability of ZnO thin films was measured from the contact angle θ (CA) of water droplets onto each ZnO film surface. After completing the UV irradiation, the water contact angle was measured on the irradiated surface by using a water droplet (~3 μL) and with a digital camera to record the droplet photos, and the average error in water contact angle measurement influenced by image quality and the built-in curve-fitting function was estimated to be ±1 degree. The UV light onto each ZnO sample was irradiated by 1520 mW/cm^2^ UV-LED light with a wavelength of 365 nm, and all the ZnO nanostructured films were stored in dark surroundings under air ambient after irradiated with UV light.

## Additional Information

**How to cite this article:** Chi, P.-W. *et al*. Internal stress induced natural self-chemisorption of ZnO nanostructured films. *Sci. Rep.* **7**, 43281; doi: 10.1038/srep43281 (2017).

**Publisher's note:** Springer Nature remains neutral with regard to jurisdictional claims in published maps and institutional affiliations.

## Supplementary Material

Supplementary Information
