# Supplementary material for: Internal stress induced natural self-chemisorption of ZnO nanostructured films
Source: Sci Rep. 2017 Feb 24;7:43281. doi: 10.1038/srep43281 (PMC5324092; doi:10.1038/srep43281)
Supplement: Supplementary Information [file srep43281-s1.doc]

**Supplementary information**

Internal stress induced natural self-chemisorption of ZnO nanostructured films

Po-Wei Chi^†^, Chih-Wei Su^†^, Da-Hua Wei^*,†^

^†^ Institute of Manufacturing Technology and Department of Mechanical Engineering, National Taipei University of Technology (TAIPEI TECH), Taipei 10608, Taiwan

**Corresponding Author**

*E-mail: [dhwei@ntut.edu.tw](mailto:dhwei@ntut.edu.tw)

**Tunable internal stress**

The internal stress/strain state of ZnO nanostructured films was manipulated by varying RF powers of 75, 100, 125, 150, 175 and 200 W, and these thin films were named as Z75, Z100, Z125, Z150, Z175 and Z200, respectively. The deposition rates of each RF power were 3.8, 4.3, 5.6, 6.5, 7.2 and 7.8 nm/min, respectively. All samples are with a total nominal thickness of 300 nm onto glass substrates without any buffer layer. Figure S1 shows the relationship between surface wettability and the internal stress of the samples. It can be clearly observed that samples with hydrophobicity occurred at the internal stress state is in compressive stress state. On the other hand, while the internal stress state is at nearly stress-free, the samples show hydrophilicity. Above results indicated that two kinds of internal stress state can be produced and effectively leaded different wetting property on the thin film surface.

**
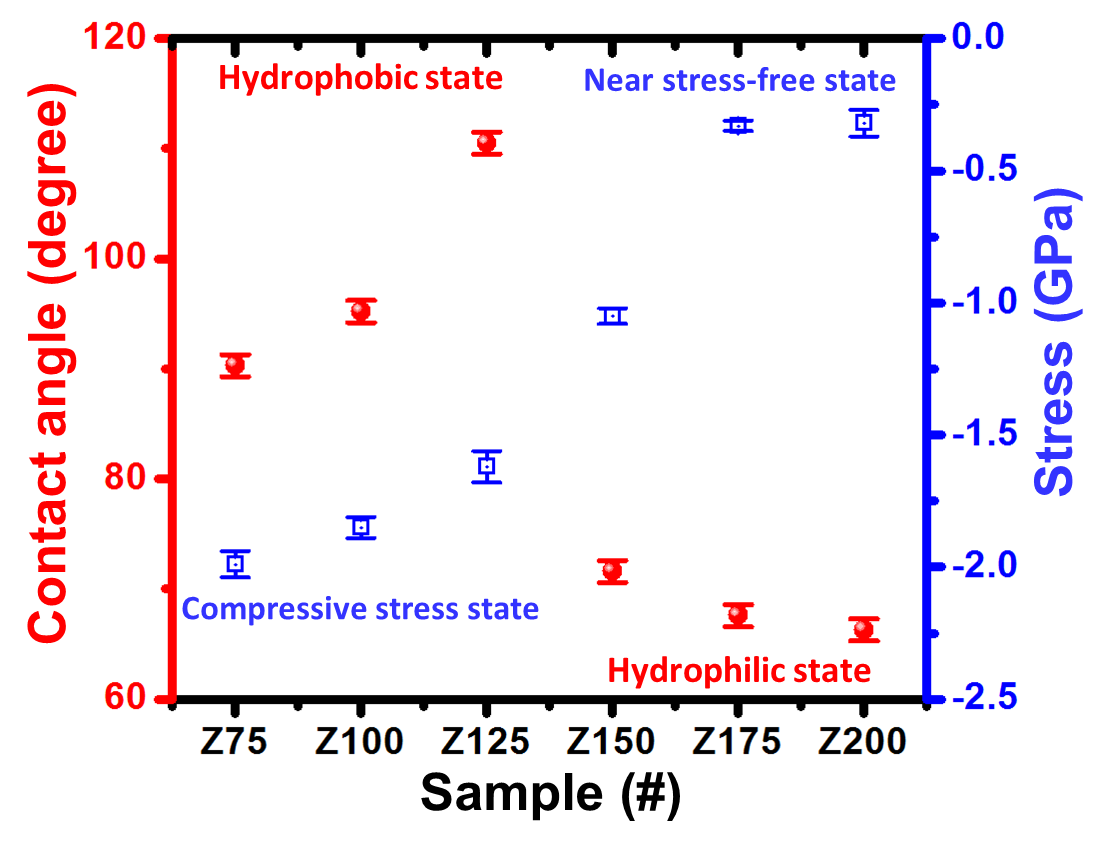
**

**Figure S1.** The plots of contact angle (red dot) and internal stress (blue dot) for all designed samples.

**Statistical study of AFM**

The line profiles of the AFM cantilever for samples Z125 and Z175 are shown in Figure S2(a) and S2(b). It can be seen that the aperture size in fraction of sample Z125 as shown in Figure S2(a) ranged from 37.3 to 48.1 nm, with an average size of 42.7 ± 5.4 nm, and the aperture size in fraction of sample Z175 as shown in Figure S2(b) ranged from 64.5 to 108.9 nm, with an average size of 86.7 ± 22.4 nm, respectively.


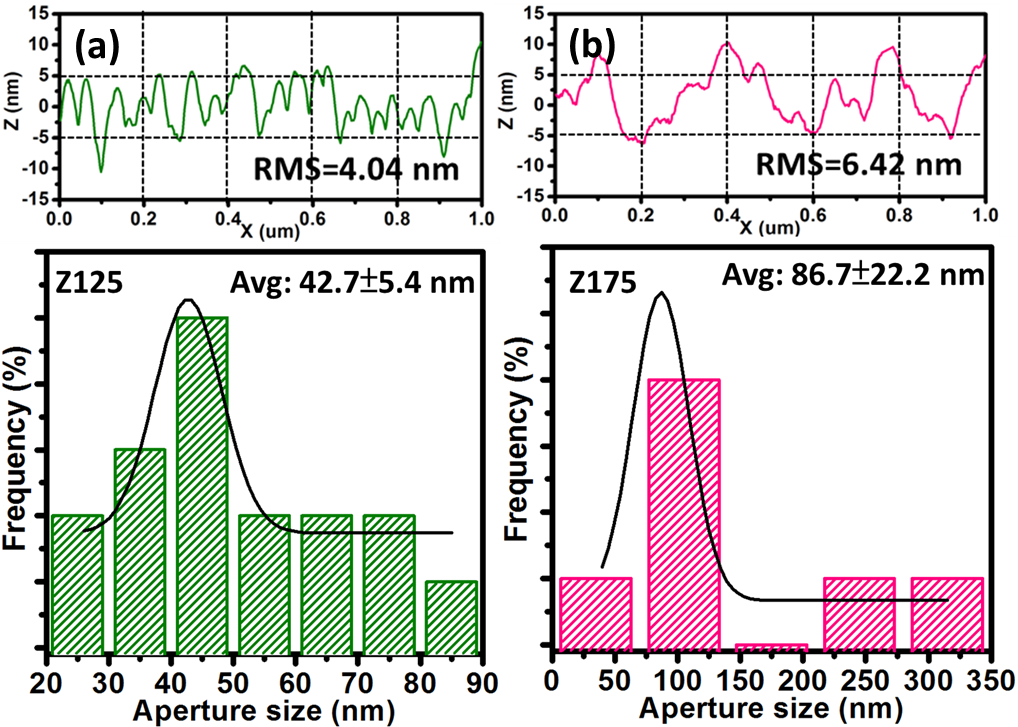


**Figure S2.** The line profiles of the AFM cantilever and the corresponding apertures size histograms for evaluating average size and its distribution of the samples Z125 (a) and Z175 (b), respectively.

**Theoretical fitting**

Generally, the band gap energy between conductive band and valence band broadens in nanosystems of semiconductor material, also this kind of phenomenon accompanies with some unique properties in electronics and optoelectronics, as compared with bulk material. Due to quantum-size confinement effect, Chen *et al.* reported the blue-shift of ZnO nanorod [1]. A similar result also has been observed in ultrathin ZnO nanotube by Yang *et al.* [2]. However, the emission by the different grain size will have the similar quantum-size confinement effect as mentioned in the report before and can be described by the following equation:


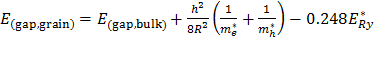
 (1)

where *h* is the Planck’s constant, *R* is the radius of ZnO grain,
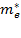
 and
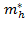
 are the effective masses of electron and hole, as purposed by Beni *et al.* [3] and Tan *et al.* [4], the effective masses of electron and hole are taken
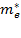
 = 0.24*m*_0_ and
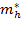
 = 2.31*m*_0_, respectively, *E*_(gap, bulk)_ is the bulk ZnO band gap (3.37 eV), and
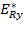
 is the exciton-binding energy (60 meV [5]). Based on Eq. 1, the relationship between the grain size and band gap of ZnO nanostructure films can be obtained as shown in Figure 5(b) (in the manuscript).

**Reference**

1. C. W. Chen *et al.* *Appl. Phys. Lett.* **88**, 241905 (2006).
2. P. H. Yang *et al. Nanoscale* **4**, 5755 (2012).
3. G. Beni *et al. Phys. Rev. B* **18**, 768 (1978).
4. S. T. Tan *et al.* J. Appl. Phys. **98**, 013505 (2005).
5. Y. W. Heo *et al.* *Mater. Sci. Eng. R* **47**, 1 (2004).
